# Combined Acoustic Emission and Digital Image Correlation for Early Detection and Measurement of Fatigue Cracks in Rails and Train Parts under Dynamic Loading

**DOI:** 10.3390/s22239256

**Published:** 2022-11-28

**Authors:** Alexander Machikhin, Anton Poroykov, Vladimir Bardakov, Artem Marchenkov, Daria Zhgut, Milana Sharikova, Vera Barat, Natalia Meleshko, Alexander Kren

**Affiliations:** 1 Moscow Power Engineering Institute, 14, Krasnokazarmennaya Str., 111250 Moscow, Russia; 2 Scientific and Technological Center of Unique Instrumentation, Russian Academy of Sciences, 15, Butlerova Str., 117342 Moscow, Russia; 3 INTERUNIS-IT, POB 140, 20B, Shosse Entusiastov, 111024 Moscow, Russia; 4 Institute of Applied Physics, National Academy of Sciences, 16, St. Akademicheskaya, 220072 Minsk, Belarus

**Keywords:** acoustic emission, digital image correlation, fatigue cracks, crack detection, dynamic loading, non-destructive testing

## Abstract

Fatigue crack in rails and cyclic-loaded train parts is a contributory factor in multiple railroad accidents. We address the problem of crack detection and measurement at early stages, when total failure has not yet occurred. We propose to combine acoustic emission (AE) testing for prediction of crack growth with digital image correlation (DIC) for its accurate quantitative characterization. In this study, we imitated fatigue crack appearance and growth in samples of railway rail and two train parts by cyclic loading, and applied these two techniques for inspection. Experimental results clearly indicate the efficiency of AE in the early detection of fatigue cracks, and excellent DIC capabilities in terms of geometrical measurements. Combination of these techniques reveals a promising basis for real-time and non-destructive monitoring of rails and train parts.

## 1. Introduction

Fatigue fractures of rails, wheelsets, solid-rolled wheels, side frames of wagon bogies, and other parts of the rolling stock frequently trigger railway accidents and incidents [1,2,3]. Despite the wide range of non-destructive testing (NDT) techniques applied in regular inspection of these parts, early detection and quantitative characterization of fatigue cracks is still an important task. Complex geometric shapes, thermal loading, subjectivity of decision-making, and other factors significantly complicate the analysis and prediction of fatigue crack growth in the parts of the rolling stock [4,5,6]. Conventional NDT methods include ultrasonic, eddy current, magnetic, and X-ray, utilize the signals reflected from the local inhomogeneities, and, thus, have relatively low sensitivity in terms of complex-shaped crack-like defects [7,8,9].

Unlike these methods, acoustic emission (AE) testing is a technique based on the analysis of elastic waves generated by the defects initiation and growth. In comparison with conventional NDT methods, AE testing has a number of advantages. It is insensitive to the shape and orientation of the defects, does not require spatial scanning, and thus is well suited for in situ structural health monitoring [10,11]. AE sources include plastic deformation, initiation and growth of cracks, fracture, corrosion, leakages, fiber debonding, etc. [10]. AE technique is widely used and well-proven in detecting the processes of fatigue cracks initiation and growth both in metallic and composite materials [12,13,14,15,16].

AE testing has been successfully applied to fatigue crack detection in rails under operational loads [17,18]. The development of advanced AE data processing algorithms significantly expands the scope of its applications for testing and detection of fatigue cracks in rails. Furthermore, in [19,20] authors used wavelet analysis to extract the signals from even small fatigue cracks hidden below the background noise. Deep learning and neural network processing enabled detection and localization of the defects in the head, web, and foot of the rail [21]. Regarding the parts of the rolling stock, AE testing exhibits high efficiency in detection and monitoring fatigue cracks in full-scale railway axles [22,23,24]. AE technique, combined with an automated loading system, has also been successfully applied to fatigue crack detection in molded pieces of rolling stock, such as the side frame and bolster of railway bogies [25,26].

In addition to identification of the defects and their growth prediction, it is important to measure their size and location. For this purpose, AE testing has to be coupled with a well-established method for geometrical measurements. One of the most promising techniques for quantifying defects in combination with AE testing is digital image correlation (DIC).

DIC is a non-contact full-field optical technique for deformation diagnostics and quantitative assessment of surface defects with high spatial resolution and accuracy [27]. It is widely in use to verify various NDT methods: ultrasound, eddy current, etc. [28,29,30]. DIC is also an effective complement to AE testing of concrete [31], reinforced concrete [32,33], metals [34,35], welded joints [36], etc. DIC may be used to confirm the cracking point determined by the AE testing [34,35], as well as identification of different damage mechanisms (plastic deformation, crack initiation, and propagation) based on comparison AE and DIC data [36,37,38,39]. DIC is also suitable for full-scale testing, including railway equipment [40], but this application is still limited due to limited field of view, specific calibration, etc.

In this study, we propose to implement DIC after primary information on the presence and location of defects is collected by AE testing, either in lab or out-of-lab environment. Further characterization and quantification of defects must be carried out by DIC under conditions close to operation. The proposed approach is thus a compromise between possible qualitative determination of defects in the field conditions and high-precision defect measurement under laboratory conditions.

A combined AE–DIC approach might be especially effective for monitoring the rolling stock parts due to their large dimensions, shape complexity, and operation under cyclic loading. In this study, we attempt to validate it by detection and quantitative characterization of fatigue cracks in samples of rails, wheelsets, and traction clamps.

## 2. Experimental Protocol

Acronyms and symbols introduced in the manuscript and their definitions are presented in Table 1.

### 2.1. Samples Preparation

For our experiments, we prepared samples from three railway parts: rail, wheelset, and traction clamp. The rail was made of E76F high-carbon steel and experiences mainly compressive stresses from the train. The wheelset, together with two steel wheels, forms part of the wagon cart. The wheelset material experiences cyclic bending stresses. The wheelset was made of OsV carbon steel. A traction clamp is necessary to connect wagons to each other. During operation, this element experiences mainly cyclic tensile and bending loads when the train starts moving. The traction clamp was made of low-alloy structural steel 20GL. The chemical composition of these materials presented in Table 2.

**Figure 1:**
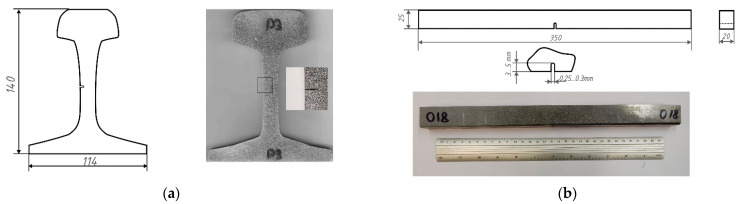

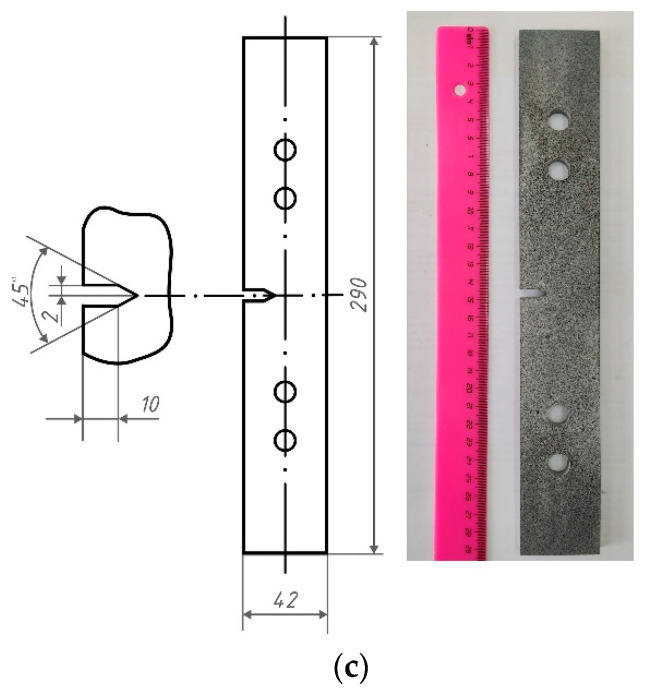
Schemes and photos of the samples made from rail (**a**), wheelset (**b**), and traction clamp (**c**).

Samples from the rail (3 pcs) were sections 10 mm thick (Figure 1a). U-notches of 0.25 mm width and 2 mm depth were produced in its web part by wire electrical discharge machining (EDM), since this does not require additional hardening of the metal surface. To make a notch of the required depth with a radius at the top of 0.25–0.3 mm, we applied a wire of 0.25 mm diameter. Samples cut from the wheelset (3 pcs) were 20 × 25 × 350 mm bars with notches of 3–5 mm depth and 0.25–0.3 mm radius, produced by EDM (Figure 1b). Samples made from the traction clamp (3 pcs) were 3 mm thick plates with lateral notches, obtained with EDM by wire cutting along a defined contour (Figure 1c). As polished metal does not have the high-contrast surface texture necessary for digital image correlation (DIC) method application, we created a random pattern on one of the samples’ surfaces using white and black spray paint.

### 2.2. Experimental Setup

**Figure 2:**
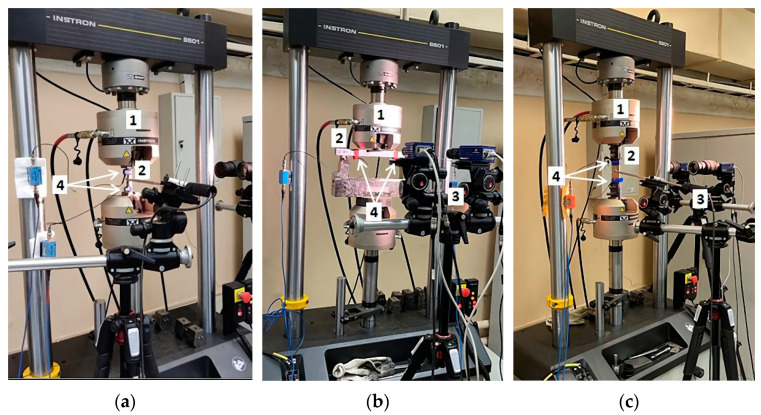
Experimental setup with rail (**a**), wheelset (**b**), and traction clamp (**c**) samples under inspection: 1, loading device (punch); 2, inspected sample; 3, DIC system; 4, AE sensors.

The experimental setup was based on the servo-hydraulic fatigue testing system Instron 8801, which was necessary to imitate cyclic loading caused by the train movement. The loading scheme was designed in order to imitate the loads that studied parts experience during operation, i.e., the rails were subjected to compressive load, the samples from the wheelset to three-point bending, and the samples from the traction clamp to tensile loading. We fixed the rail samples using grips (Figure 2a). Wheelset samples were placed on a three-point static bending fixture (Figure 2b), including two lower supports separated by 250 mm distance, and an upper cylindrical punch with a diameter of 10 mm. Samples from the traction clamp were installed with grips through damping pads (Figure 2c).

**Figure 3:**
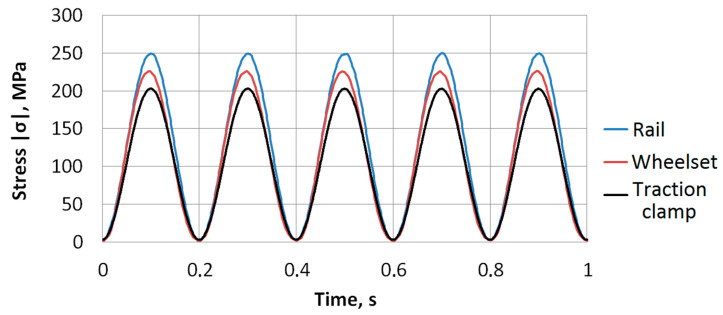
Loading cycles applied to the inspected samples.

Figure 3 shows fatigue stress cycles applied to the samples. Sinusoidal loading was necessary to ensure the smooth running of the punch in the testing machine when reaching the minimum and maximum values of the cycle. This reduced the level of acoustic noise that occurred in the testing machine when passing through the extreme points of the cycle, thus minimizing the amplitude threshold for the recorded AE signals. Maximal stress was limited by the yield strength of the material *σ_y_* at the level (0.48…0.72)*σ_y_*. This is close to the values of maximum allowable stresses that may occur during operation of the inspected elements. Under such stress, in the absence of notches and cracks, the structural elements stay in the elastic region during operation. In the presence of cracks, or severe stress concentrators, plastic deformation and subsequent destruction occur with high probability. The loading frequency 5 Hz was a good compromise between the duration of the experiment, the acoustic noise level, which increases in the testing machine with the raise of the loading frequency, and the duration of the tests. Loading conditions applied to the samples are specified in Table 3. Loading was accompanied with continuous recording of DIC and AE data.

We installed two AE sensors GT200 (GlobalTest) with a resonant frequency of 180 kHz on each sample, symmetrically, with respect to the stress concentrator, and connected them to the preamplifier with 26 dB gain. To achieve acoustic contact between the AE sensors and the sample, we used Lithol-24 as an intermediate layer. The AE system A-Line 32D PCI (INTERUNIS-IT) acquired the acoustic signals with a frequency range of 100–400 kHz.

For DIC measurements, we used the machine vision system StrainMaster (LaVision). It consists of PC with DaVis 8.4 software (LaVision GmbH, Göttingen, Germany), a synchronization unit, and two monochrome cameras Imager SX (2/3”, 2456 × 2058, 12 bit) equipped with lenses Sigma (*f* = 18–200 mm, 1:3.5–6.3). Each experiment included 2400 × 1300 image acquisition of a 50 × 40 mm sample’s notched area at 20 fps, their joint processing by DIC algorithms, and calculating the strain maps *ε* (*x,y*).

### 2.3. Data Processing

To determine the AE signals caused by fatigue crack’s initiation and growth, we used linear location of AE sources and placed AE sensors at the edges of the samples to be able to locate AE sources within the maximal area. The distance between them was 50 mm for the rail, 160 mm for the wheelset, and 60 mm for the clamp. The linear location algorithm provided the position *l* of the AE source located on the line between AE sensors. Dependence of the number of AE events *N* on *l* vividly illustrates such an approach. Due to typical sound velocity, 3000 m/s, in the inspected steels and sampling step 1 μs, the inaccuracy *dl*_AE_ of measured *l* was about 3 mm. Therefore, the distance between AE sensors for linear location algorithm was divided into 3 mm intervals. This was rather coarse for accurate measurements, but was enough for navigating a fine tool with higher accuracy to the area of the crack for detailed metrological inspection. To identify the moment *T*_AE_ of crack appearance, the threshold criterion [41] was applied. For each new AE event, we evaluated its position and updated the location histogram. Then, the ratio of the location maximum *N*_max_ of histogram to the mean value *N*_mean_ over all intervals, except for the maximum, was calculated. A crack was considered to be detected when the maximum *N*_max_ in the histogram was *η_T_*_AE_ times greater than the mean value *N*_mean_, i.e.,

*N*_max_*/N*_mean_ ≥ *η_T_*_AE_, (1)

**Figure 4:**
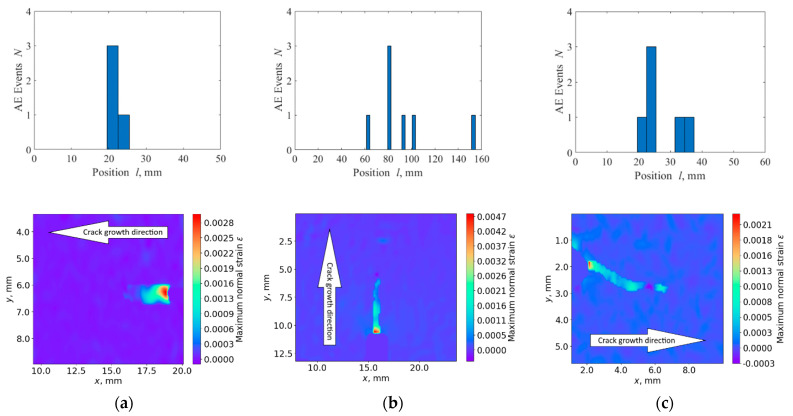
AE (*N*(*l*), upper row) and DIC (*ε*(*x*,*y*), lower row) data for rail (**a**), wheelset (**b**), and traction clamp (**c**) samples at the moments *T*_AE_ and *T*_DIC_, respectively.

The upper row of Figure 4 shows the histograms *N*(*l*) for all three types of the inspected samples at the moment *T*_AE_ when *η_T_*_AE_ = 3. At this empirical threshold, it was possible to distinguish a clear maximum. Its location corresponds to the crack position in the experiments. We can see that this threshold corresponds well to the signal level well above the emissivity of the inspected materials.

The DIC method was necessary to obtain information about the deformation of the samples during their mechanical tests. It was based on the comparison of the images of the sample surface. The first (reference) image was taken before the test. In this image, the surface is considered to be deformation-free. The second image was captured during the test, when the sample was loaded. Quantifying its difference, with respect to the reference, provided an estimate of the deformation that the sample experienced under loading.

In this study, we used the least squares matching algorithm, together with the region grow algorithm. First, initial (seeding) points on the surface of the sample were set. For them, an affine transformation of a small area (interrogation window) around the point in the original image was searched. After matching was achieved, similar search was performed for neighboring areas, remote from the original one by a given step. The starting approximation obtained for the initial point was necessary to find their affine transformation. Thus, the calculation area increased iteratively with respect to each initial point. The affine transformation allows estimating the sample surface deformation in pixel units and recalculating them into real dimensions (mm/μm) using the camera calibration data.

User-defined inputs for image processing with DIC were the size of the interrogation window, the step for splitting into windows, and the coordinates of the initial points. There were also additional settings for adjusting the speed-to-precision ratio, mainly affecting the intermediate filtering of the affine transforms. The main parameter for processing was the size of the interrogation window. Its assignment determines the ratio of spatial resolution and measurement error. Smaller windows lead to a higher spatial resolution, but also to a higher uncertainty. Processing used a window size of 29 to 35 pixels depending on the sample, with a step size of 4 to 8 pixels. Typical error for a window size of 31 pixels is 0.005 pixels.

To determine appearance of the crack and evaluate its dimensions from DIC data, we implemented the technique based on maximum normal strain measurement from displacement vector fields [42]:

(2) $$\varepsilon =\frac{\varepsilon_{x}+\varepsilon_{y}}{2}+\sqrt{{\left(\frac{\varepsilon_{x}-\varepsilon_{y}}{2}\right)}^{2}+\gamma_{xy}^{2}}$$

where *ε*_x_ = d*V_x_*/d*x* and *ε*_y_ = d*V_y_*/d*y* are deformations in *x* and *y* directions, *V_x_* and *V_y_* are displacements detected by DIC*,* and *γ*_xy_ = (d*V_x_*/d*y* + d*V_y_*/d*x*)/2 is shear strain.

Processing the scalar normal strain fields includes binarization using Otsu thresholding algorithm and skeletonization. This enables accurate segmentation of the crack area. To determine the moment of crack initiation *T*_DIC_, we calculated the maximum normal strain *ε* in this area and compared it with the threshold *ε_T_*_DIC_ defined by the transition from elastic deformation to plastic in the notch area [43]. If we neglect the small value of material’s plastic deformation at this level, then the threshold value *ε_T_*_DIC_ is close to the ratio *σ_y_/E*. From tensile tests of the studied materials, the values of the yield stress are *σ_y_* ≈ 520 MPa for the rail, *σ_y_* ≈ 320 MPa for the wheelset, and *σ_y_* ≈ 360 MPa for the clamp. The elastic modulus for all materials is *E* ≈ 210,000 MPa. Thus, the resulting threshold values are *ε_T_*_DIC_ = 0.0025 for the rail, *ε_T_*_DIC_ = 0.0015 for the wheelset, and *ε_T_*_DIC_ = 0.0017 for the traction clamp. As soon as we have

*ε* > *ε_T_*_DIC_, (3)

we assume the crack to be detected. The lower row of Figure 4 shows typical strain maps *ε*(*x*,*y*) calculated at the moment *T*_DIC_ for all three inspected samples. In these distributions, maximum normal strain *ε* is equal to the threshold value *ε_T_*_DIC_. We may see that this criteria is quite reliable in terms of crack detection, but the DIC system needs accurate positioning, and, thus, has to be navigated to the area of the crack by preliminary AE measurements. This is the basis of the proposed two-stage approach to the detection and quantitative evaluation of fatigue cracks.

## 3. Results

**Figure 5:**
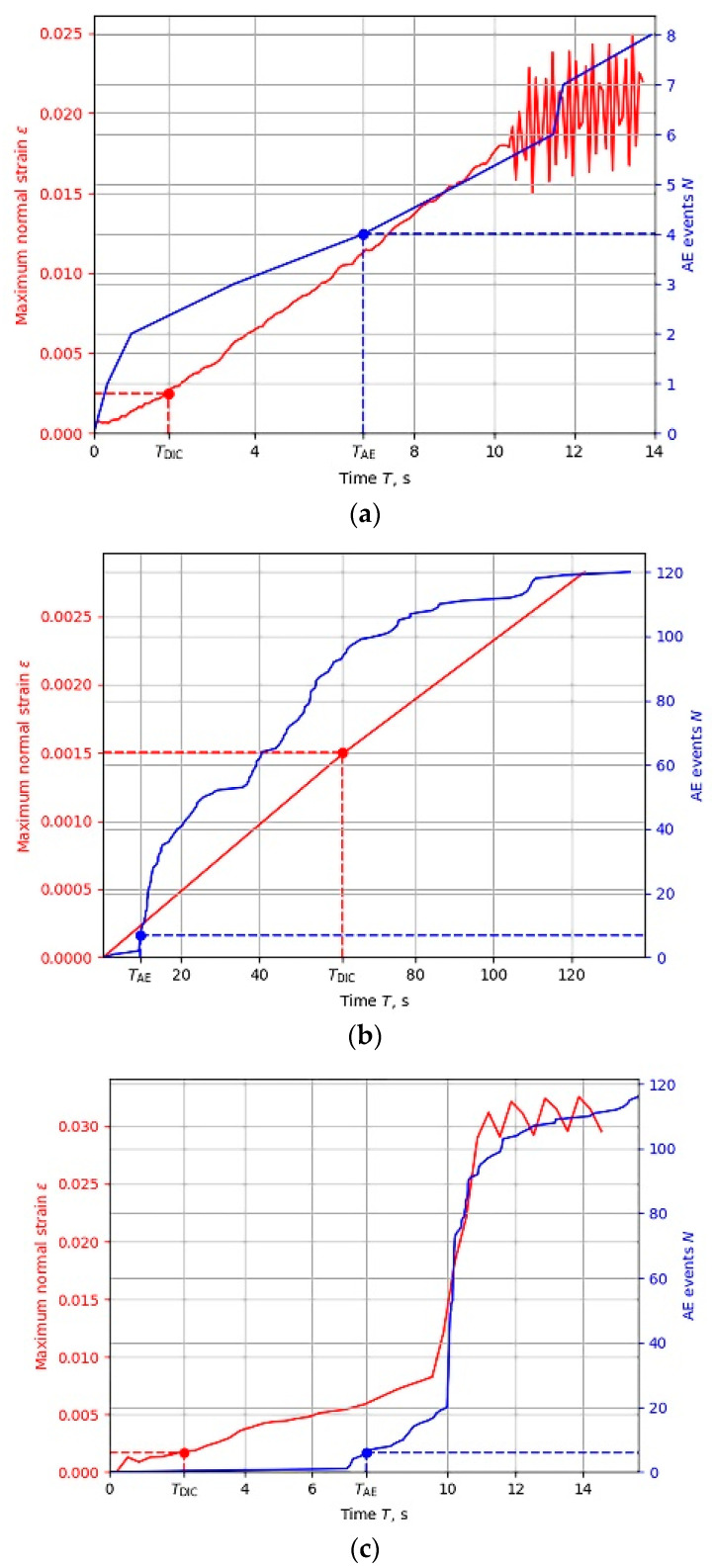
Temporal dependencies of AE (blue) and DIC (red) data in rail (**a**), wheelset (**b**), and traction clamp (**c**) samples.

To validate this approach, we applied the described experimental and data processing techniques to all nine samples. Each experiment included continuous acquisition of AE and DIC data, evaluation of AE events number *N* and maximum normal strain ε, verification of criteria (1) and (3) fulfillment, and measurements of crack location and dimension. Figure 5 shows the typical temporal dependences of *N* and *ε* for all three types of inspected samples. Time interval of data acquisition covers the moments *T*_AE_ and *T*_DIC_ of fatigue crack appearance according to (1) and (3), as well as its further growth.

**Figure 6:**
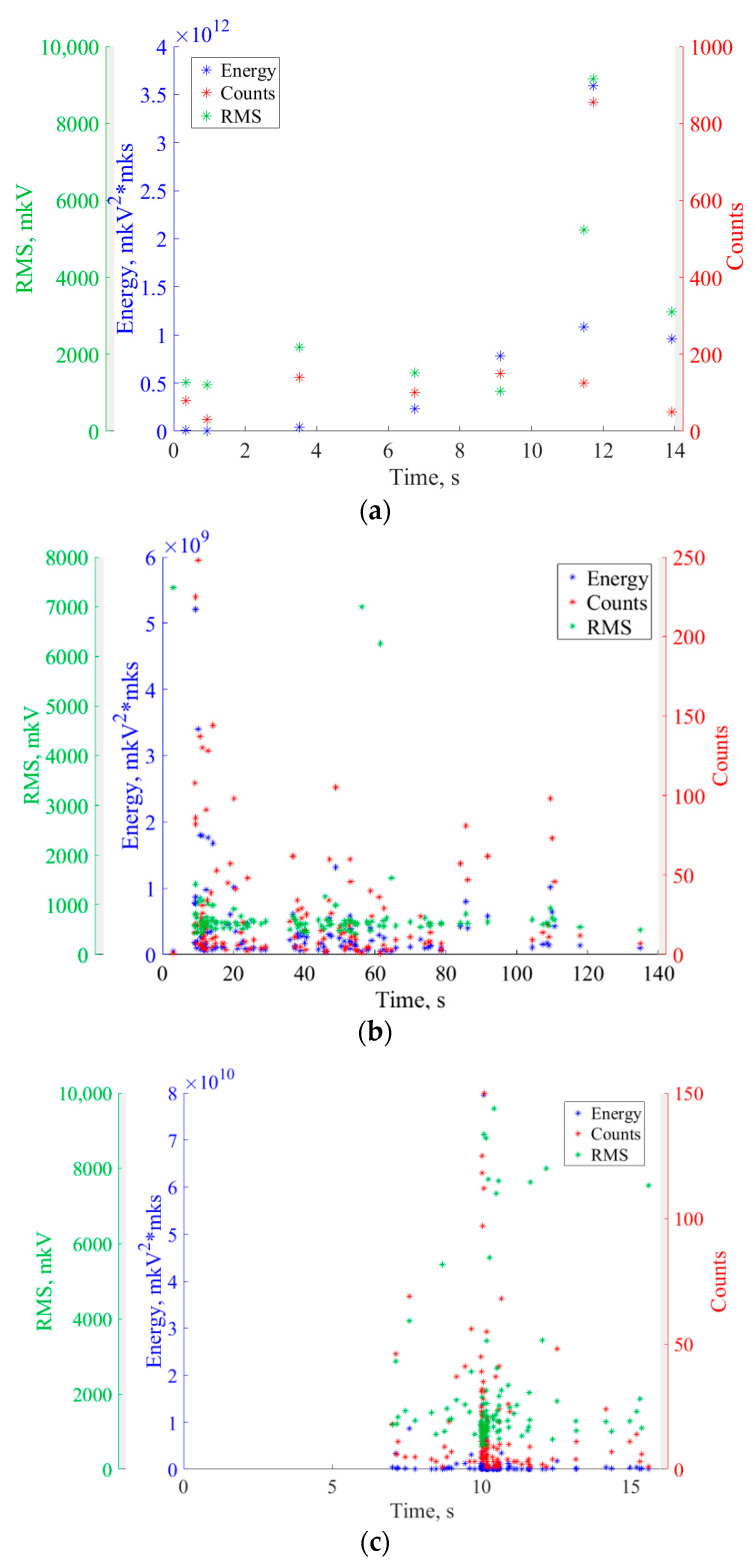
Temporal dependencies of AE parameters in rail (**a**), wheelset (**b**), and traction clamp (**c**) samples.

Temporal dependencies of AE events have different shapes that depend on the samples and materials, and correspond to the accumulation of damage processes in the samples. At the moment *T* < *T*_AE_ preceding crack detection by AE testing, not all samples demonstrate a sharp increase in AE events, which corresponds to the appearance of AE source by the destruction process [34]. For a detailed analysis, more AE parameters (energy, counts, and RMS) of recorded events were considered (Figure 6).

It is well known that a sharp increase in these parameters indicates a crack initiation [44,45,46,47]. In all cases, we see such increases. In the wheelset sample, a sharp increase in AE parameters (Figure 6b) coincides with *T*_AE_ moment (Figure 5b). In the rail and traction clamp samples, the main peak of AE parameters is observed to have a slight delay with respect to the moment *T*_AE_. This may be explained by a high sensitivity of AE testing to the registration of the early crack initiation processes. Moreover, the fact that the location determined by AE data fits well to the notch position in the samples confirms the correctness of the proposed criterion.

In contrast to *N*(*T*), temporal dependence of strain *ε*(*T*) increases monotonically. Its shape does not show definitely the moment *T*_DIC_ of crack appearance. This moment is, to a large extent, defined by the threshold *ε_T_*_DIC_. That is why we may see that the moment *T*_AE_ does not always precede *T*_DIC,_ as we might expect (Figure 5b), but *T*_DIC_ may be smaller than *T*_AE_ (Figure 5a,c). Thus, unlike the AE technique, DIC does not enable objective and evident indication of the moment of fatigue crack appearance, but may provide an objective measure of the crack’s geometrical parameters.

We should note that the moments of crack appearance vary depending on the material. In the rail, for example, a crack appears quite early, though the loading is applied in the compression mode with a relatively small cycle load 0.48·*σ_y_*. According to AE data in Figure 5a, a crack appears at *T*_AE_ ≈ 7 s. For comparison, in the wheelset material (Figure 5b), loaded more at 0.7·*σ_y_* level, 3-point bending causes the appearance of tensile stress at the notch tip, and the crack formation time from AE data is somewhat longer (*T*_AE_ ≈ 10 s). This may be caused by differences in the mechanical properties of these materials. Despite higher strength characteristics, the rail material has low ductility, which makes the rail material less resistant to crack initiation and development.

**Figure 7:**
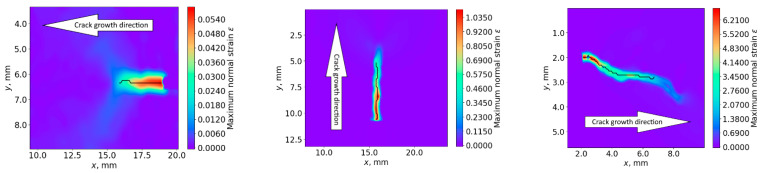

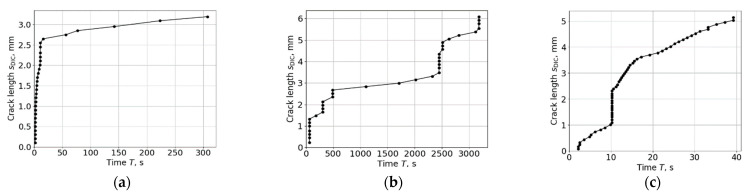
Field of crack maximum normal strain (**upper row**) and temporal dependencies of the calculated crack length (**lower row**) for rail (**a**), wheelset (**b**), and traction clamp (**c**) samples.

To illustrate the metrological capabilities of DIC, we measured the crack’s dimension *s*_DIC_ as a length of skeletonized line obtained after processing the field of maximum normal strain *ε*(*x*,*y*) (see upper row of Figure 7) and plotted its temporal *s*_DIC_(*T*) dependence from the moment *T*_AE_ (see lower row of Figure 7). Inaccuracy *ds*_DIC_ of this measurement, as well as the inaccuracy of crack’s position estimation by DIC, may be estimated as

*ds*_DIC_ = *dl*_DIC_ = (*L*⋅*Δ*/*f*)*dn*, (4)

where *β* = *f/L* is the linear magnification of DIC optical system, *L* is the distance to the inspected sample, *f* is the focal length of the lens, *Δ* is the pixel pitch of the camera, and *n* is the measured crack’s length in pixels. 

If we measure the length with inaccuracy *dn* = 1 pixel, then equation (4) may be simplified: *ds*_DIC_ = *dl*_DIC_ = *Δ*/*β*. With respect to our experiments (*β* = 0.17, *f* = 170 mm, *L* = 1 m, *Δ* = 3.45 μm), this value is about 0.02 mm, i.e., much smaller than the *dl*_AE_ = 3 mm measured by AE.

## 4. Discussion

**Figure 8:**
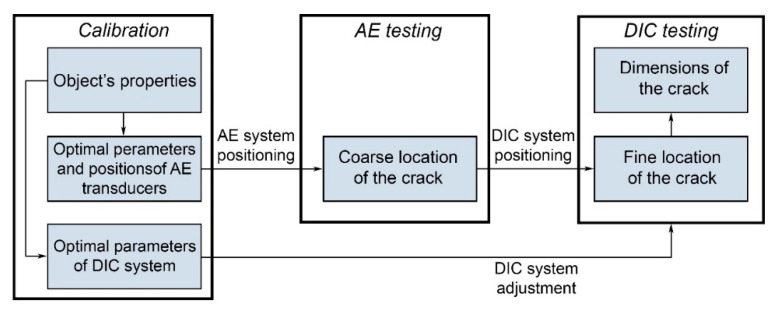
Proposed protocol for AE–DIC inspection.

In this study, we propose a coarse-to-fine approach to fatigue crack detection and measurement in the railway and train parts. It consists of two stages: AE and DIC testing (Figure 8). For such a technique, accurate in-house calibration of both AE and DIC systems is necessary (see Section 2). First of all, analysis of the sample’s material and shape must be carried out to set the criteria (1) and (3) for AE and DIC, respectively. To set the criterion (1), one needs to estimate acoustic emissivity of the inspected samples. Proper positioning of AE sensors and minimizing the location error *dl*_AE_ require measurements of sound velocity and sensitivity of the AE sensors [48]. To set criterion (3), *ε_T_*_DIC_ value needs to be defined. Based on *dl*_AE_ and available DIC system, we may calculate the optimal parameters *β* and *f* of the optical system. For proper strain measurements across the whole image, the DIC system also needs advanced geometrical calibration [49].

After the calibration stage, AE sensors must be installed on the inspected part for monitoring a fatigue crack’s appearance and coarse evaluation of its location. If criterion (1) is fulfilled, the DIC system may be guided to the calculated position of the AE source. Immediate navigation of DIC system to this area allows fine measurement of the crack’s location, real-time tracking of its geometrical features, and damage prevention.

The key advantage of the proposed AE–DIC approach is the early detection of defects in railway infrastructure components. This enables studying the defect growth dynamics in real time using the DIC technique. In this case, the investigated sample will be in its natural environment. Thus, it is possible to obtain unique experimental data on the evolution of defects specific to normal operation. This data can significantly improve the theoretical models used in assessing the impact of loads on the studied components, and can be used to develop new criteria for the early detection of defects in these components. The proposed approach allows speeding up the monitoring of fatigue cracks due to the localization of AE source and applying DIC specifically in this area. It is not necessary to stop loading for verifying AE sources, due to non-contact and real-time DIC measurements. 

In the railway industry, this approach may be useful for fatigue testing of full-scale railway and train parts as a part of automated loading systems for real-time fatigue crack detection and quantification. It might be efficient and reliable for the samples of various materials and shapes. Though we verified it for railway and train parts, we believe it will have many more applications in multiple fields. For better performance, this technique may be supplemented with mathematical modeling [50] or any other fatigue crack detection methods [51].

## 5. Conclusions

We proposed a two-stage approach to NDT inspection of rails and two train parts under cyclic loading. It is based on sequential application of AE and DIC techniques, and is suitable for real-time evaluation. 

We introduced AE criterion as the ratio of the location maximum *N*_max_ in the histogram *N*(*l*) to the mean value *N*_mean_ over all intervals, except for the maximum. This value provides both fatigue crack detection and its coarse location. DIC enables verification of the defect presence and refinement of its position. The DIC criterion for defect detection is reaching the normal strain threshold value *ε_T_*_DIC_.

Experimental results clearly indicate the efficiency of AE for early detection and coarse location of fatigue cracks, and excellent DIC capabilities in terms of its geometrical measurements. In practice, AE might be applied for timely notification of crack appearance and guidance of the DIC system to the area of the crack for quantitative evaluation. The combination of these techniques reveals a promising basis for real-time and non-destructive monitoring rails and train parts.

## Figures and Tables

**Table 1:** List of acronyms and symbols used in the study.

Acronym	Description	Symbol	Description
NDT	non-destructive testing	*σ_y_*	yield strength
AE	acoustic emission	*σ*	cycle amplitude
DIC	digital image correlation	*l*	position of the AE source
EDM	electrical discharge machining	*dl*_AE_	inaccuracy of the AE source position
*ε*	maximum normal strain	*N*	number of AE events
*V_x_* and *V_y_*	displacements detected by DIC	*η_T_*_AE_	ratio of *N*_max_ and *N*_mean_, when the crack is detected
*ε_T_*_DIC_	threshold value defined by transition from elastic deformation to plastic in the notch area	*N*_max_	location maximum of histogram
*s*_DIC_	crack’s dimension as a length of skeletonized line obtained after processing the field of maximum normal strain	*N_mean_*	mean value over all intervals, except for the maximum
*f*	focal length of the lens	*T_AE_*	moment of crack appearance
*L*	distance to the inspected sample	*T*_DIC_	moment of crack initiation
Δ	pixel pitch of the camera	*ε*_x_	deformation in *x* directions
*n*	measured crack’s length in pixels	*ε*_y_	deformation in *y* directions

**Table 2:** Chemical composition of the test samples’ materials.

Part	Steel	Composition, wt. %					
		C	Si	Mn	V	S	P
Rail	E76F	0.71–0.82	0.25–0.60	0.75–1.15	0.03–0.15	≤0.025	≤0.025
Wheelset	OsV	0.40–0.48	0.15–0.35	0.55–0.85	-	≤0.045	≤0.04
Traction clamp	20GL	0.15–0.25	0.20–0.40	1.20–1.60	-	≤0.04	≤0.04

**Table 3:** Loading conditions applied to the samples.

Part	Type of Stress	Cycle	Frequency, Hz	Cycle Amplitude *σ*, MPa	Material Yield Stress *σ_y_*, MPa	*σ*/*σ_y_*
Rail	Compressive	Sinusoidal	5	250	520	0.48
Wheelset	3-point bending			230	320	0.72
Traction clamp	Tensile			200	360	0.55

## Data Availability

The data presented in this study are available on request from the corresponding author.
